# A Case of bilateral concomitant inguinal and femoral hernias treated with transabdominal preperitoneal repair

**DOI:** 10.1002/ccr3.3283

**Published:** 2020-09-08

**Authors:** Yasunori Uesato, Mitsuhisa Takatsuki

**Affiliations:** ^1^ Department of Digestive and General Surgery University of Ryukyus Nishihara Japan

**Keywords:** bilateral hernias, femoral hernia, laparoscopic surgery, Occult hernias, subclinical hernias, TAPP

## Abstract

Synchronous bilateral hernias are very rare, and subclinical hernias as this case are very difficult to diagnose preoperatively. Laparoscopic surgery, which can accurately confirm and reinforce the weakened bilateral inguinal region, was very useful.

## Graphical abstract

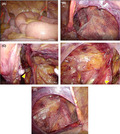

## 1 INTRODUCTION

Bilateral concomitant inguinal and femoral hernias are very rare. Preoperative examinations may not be able to detect coexisting inguinal hernias. However, laparoscopic surgery can identify these subclinical hernias and repair them appropriately. We report about the use of laparoscopic surgery to repair coexisting bilateral inguinal and femoral hernias.

Inguinal hernia repair is the most common operation performed by general surgeons.^1^ Inguinal hernias are generally diagnosed by physical findings and computed tomography (CT), but in rare cases, other hernias may be observed during surgery. If such hernias remain unobserved and unrepaired, they may result in chronic groin pain and complications including incarceration or strangulation may arise. Such occult hernias are often overlooked during open repair and may require additional postoperative treatment, particularly if located on the contralateral side. On performing inguinal hernia repair using laparoscopy, occult hernias can be easily detected and repaired during the same operation without additional incisions.^2^ Herein, we report about a case of bilateral inguinal and femoral hernias diagnosed and repaired using the transabdominal preperitoneal approach (TAPP), wherein only a single right inguinal hernia was detected by preoperative examination. This work is reported accord to the SCARE criteria.^3^

## 2 CASE REPORT

**Figure 1:**
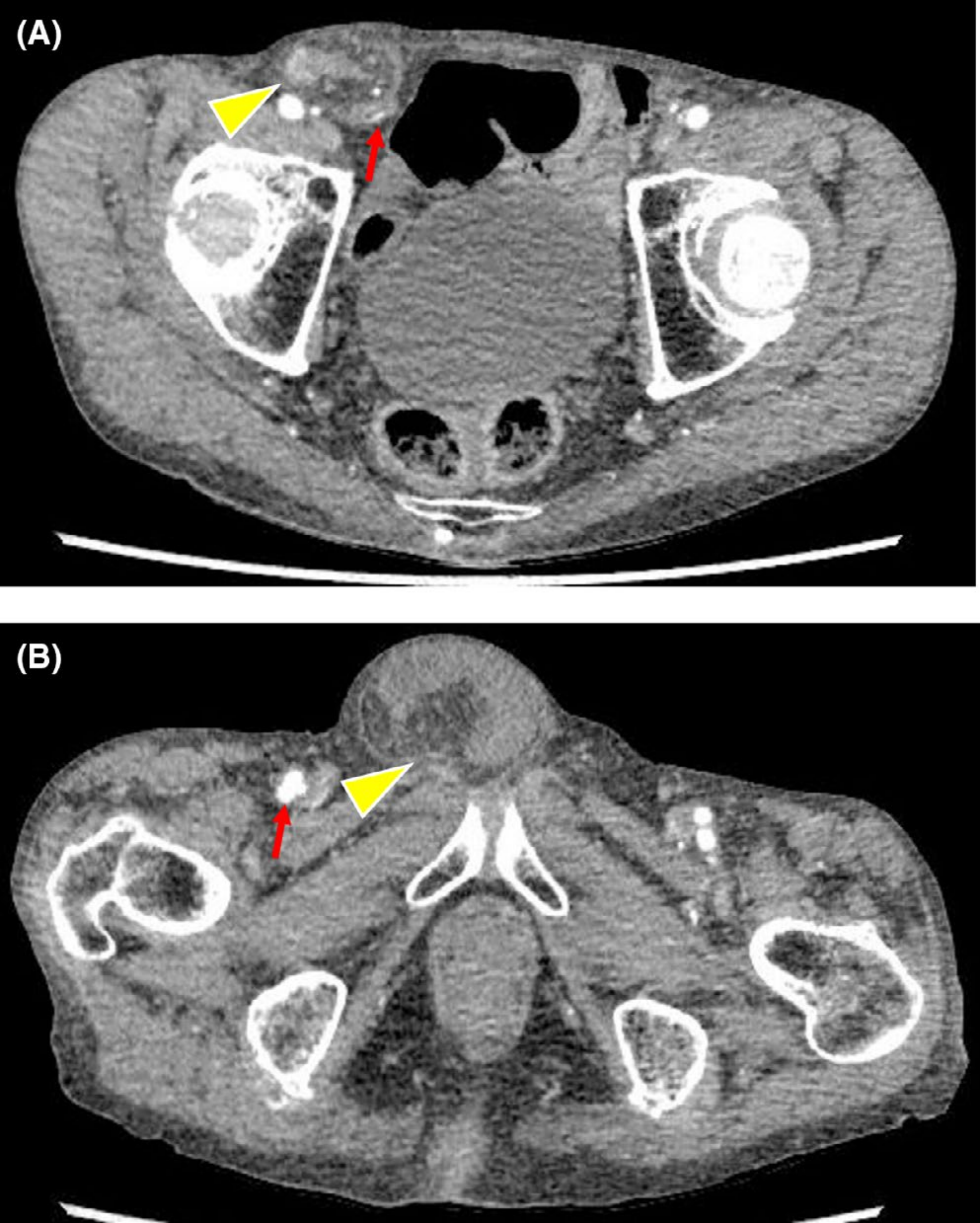
Abdominal contrast‐enhanced computed tomography findings. External inguinal hernia in the right groin (△). No obvious hernias were found in the left side. (→) indicates the lower abdominal wall artery. No obvious femoral hernia was found in the right groin. No evidence in the groin. (△) indicates inguinal hernia and (→) indicates the lower abdominal wall artery

**Figure 2:**
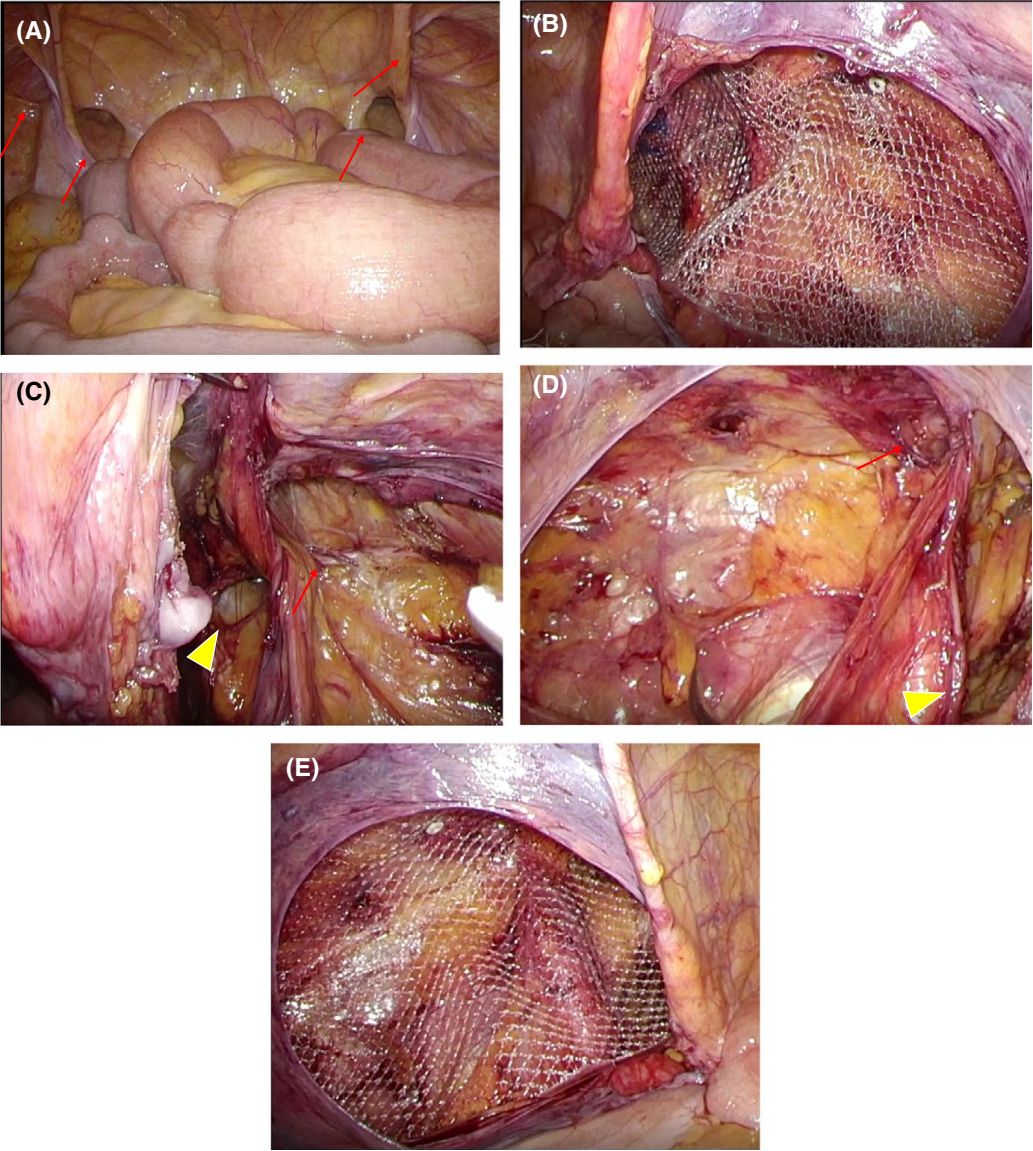
Intraoperative findings. A, Inguinal and femoral hernias are observed on both sides (→). B, An image of the right inguinal region after the preperitoneal cavity has been removed. (→) indicates inner groin ring and (△) indicates thigh ring. C, Image after placing the mesh in the right groin. It covers the inner inguinal ring, Hesselbach's triangle, thigh ring, and obturator foramen. D, An image of the left inguinal region after the preperitoneal cavity has been removed. (→) indicates inner groin ring and (△) indicates thigh ring. E, Image after placing the mesh in the left groin. It covers the inner inguinal ring, Hesselbach's triangle, thigh ring, and obturator foramen

The patient was a man in his 80s with no remarkable medical or family history. He presented at our emergency department with swelling and pain in the right groin. The right groin showed a swelling of 10 × 10 cm. Vital signs and blood tests were normal at the visit. Abdominal contrast‐enhanced CT showed prolapse of the small intestine into the right groin (Figure 1). The hernia was located outside the inferior abdominal wall artery, suggesting an indirect hernia. The size of the hernia gate was 3 × 2 cm. The left side did not show any apparent hernias. On confirming that there was no apparent intestinal ischemia, manual return was performed and the patient was hospitalized for observation. He was discharged 3 days later, after confirmation that there were no complications such as intestinal ischemia. It was agreed upon that surgery would be performed at a later date on a watch‐and‐wait basis. TAPP was used to repair it. The operation was performed in a supine position under general anesthesia. On placing the 12‐mm port in the umbilicus using the open method and examining the abdominal cavity, the coexistence of a femoral hernia as well as an indirect inguinal hernia was observed on the right side. Further observation of the left side also revealed an indirect inguinal as well as a femoral hernia (Figure 2). Although the patient was asymptomatic, surgery was also indicated for the left side, and a simultaneous repair was then performed. Two 5‐mm ports were placed in the left and right abdomen, respectively, and the operation was performed with three ports in total. The first repair was performed on the right hernia. The peritoneum was linearly incised from the outside of the hernia tract toward the hernia tract, and the space between the peritoneum and the preperitoneal fat was separated to form a space for placing the mesh. Exfoliation was performed to include the thigh ring. The inside was sufficiently dissected up to the rectus abdominis muscle, and the outside was sufficiently dissected to the right upper iliac spine. Thereafter, a Bard 3D Max Light ® mesh (10 × 15 cm) was placed and fixed to cover the inner inguinal ring, Hesselbach's triangle, and thigh ring completely. The incised peritoneum was surgically closed with 3‐0 Vicryl sutures. The left hernia was repaired using the same procedure (Figure 2). The postoperative progress of the patient was good, and he was discharged 2 days later. No complications or recurrence has been reported for 3 years since the surgery.

## 3 DISCUSSION

Groin hernias are common disorders, with a wide range of variations, and they may be difficult to diagnose preoperatively. As a result, the nature and numbers of hernias observed in surgery may differ from those identified at diagnosis. Of note, subclinical contralateral groin hernias and unsuspected femoral hernias in patients undergoing laparoscopic inguinal hernia repair are reported in 8%‐28%^4^, ^5^, ^6^, ^7^ and 7.2%‐11.1% of patients, respectively.^8^, ^9^ In the present case, a right inguinal hernia was diagnosed, but a subclinical hernia was also found on the left side during laparoscopy. If an anterior approach had been performed, the left side hernia may not have been observed, necessitating repair at a later date. The present case was an extremely rare one as simultaneous bilateral inguinal and femoral hernias were observed. Reports of three or more simultaneous hernias are very few,^10^, ^11^, ^12^ and to the best of our knowledge, this is the first report of both bilateral inguinal and femoral hernias. Strangulation or incarceration is the chief complaint for femoral hernias.^13^ In the present case, the right inguinal region was incarcerated, but the inguinal ligament protruded from the cranial side, and CT did not detect this complication in the femoral hernia. Data suggest that femoral hernias are more common in women over the age of 50,^14^ which would have made it extremely difficult to suspect femoral hernias in the present case.

Laparoscopic surgery is the standard procedure for inguinal hernia repair and its advantages include a good view of the surgical field, reduction in wound pain, and, the most remarkable aspect, easy observation, diagnosis, and repair of subclinical and contralateral groin hernias.^15^, ^16^, ^17^ In this case, three additional hernias that could not be detected preoperatively were successfully detected during laparoscopic surgery. The presence of a femoral hernia does not change the repair procedure. As in the case of the initial hernia, the repair operation is performed so that the preperitoneal space is removed, including the inner inguinal ring, Hesselbach's triangle, and thigh ring. The mesh is placed in a manner same as that used during as the repair of the noncoexisting type hernia, and the largest possible mesh is used. The wound is also similar to that incurred when repairing the noncoexisting type hernia and postoperative pain does not increase. We conclude that laparoscopic surgery offers a superior means of repairing groin hernias to the anterior approach, although the anterior approach may be the better method if solid adhesion of the preperitoneal cavity is anticipated, such as after prostate surgery. In these cases, performing a prone‐position CT scan before surgery is advisable as studies have shown that occult hernias are easier to detect with prone‐position CT than with supine position CT.^18^

## 4 CONCLUSION

Synchronous bilateral hernias are very rare, and subclinical hernias, as reported in this case, are very difficult to diagnose preoperatively. Laparoscopic surgery is particularly useful as it allows the accurate diagnosis and repair of hernias in the weakened bilateral inguinal region.

## CONFLICT OF INTEREST

None declared.

## AUTHOR CONTRIBUTIONS

All authors contributed to the acquisition and analysis of data. YU was major contributors in writing the manuscript. All authors read and approved the final manuscript.

## ETHICS APPROVAL AND CONSENT TO PARTICIPATE

Obtained from the patient in written.

## CONSENT FOR PUBLICATION

Written consent to publish was obtained for the publication of all clinical details and images, and the consent form is available for review by the editor of the journal.
